# Bis(diethyl­enetriamine-κ^3^ *N*,*N*′,*N*′′)nickel(II) bis­(1,2-dicyanoethene-1,2-dithiolato-κ^2^ *S*,*S*′)nickel(II)

**DOI:** 10.1107/S1600536808028663

**Published:** 2008-09-13

**Authors:** Dao-Peng Zhang, Hai-Long Wang, Li-Fang Zhang, Zhong-Hai Ni

**Affiliations:** a School of Chemistry & Chemical Technology, Shandong University, Jinan 250100, People’s Republic of China

## Abstract

The title compound, [Ni(C_4_H_13_N_3_)_2_][Ni(C_4_N_2_S_2_)_2_], has been synthesized by the reaction of Ni(ClO_4_)_2_·6H_2_O, diethyl­enetriamine (deta) and Na_2_[Ni(mnt)_2_] [mnt = maleonitrile­dithiol­ate(2-)] in methanol. The structure is composed of a [Ni(deta)_2_]^2+^ cation and a [Ni(mnt)_2_]^2−^ anion. The coordination geometry of the Ni^II^ ion in the cation is slightly distorted octa­hedral, defined by six N atoms from two deta ligands, while the Ni^II^ ion in the anion is four-coordinated by four S atoms from two mnt ligands in a slightly distorted square-planar geometry. The cations and anions are connected by N—H⋯N hydrogen bonds.

## Related literature

For related literature, see: Bois *et al.* (1998 ▶); Keum *et al.* (1992 ▶); Miller *et al.* (1989 ▶); Ren *et al.* (2001 ▶); Robertson & Cronin (2002 ▶); Simmons *et al.* (1962 ▶). 
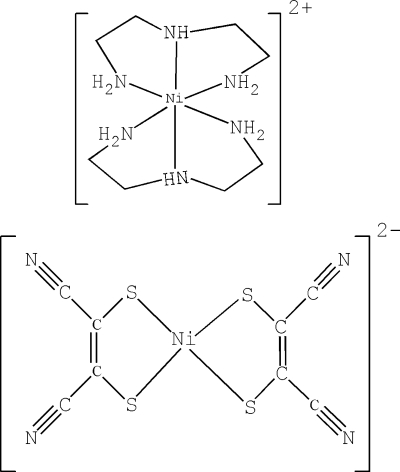


## Experimental

### 

#### Crystal data

[Ni(C_4_H_13_N_3_)_2_][Ni(C_4_N_2_S_2_)_2_]
*M* *_r_* = 604.13
Monoclinic, 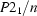

*a* = 9.589 (3) Å
*b* = 16.910 (5) Å
*c* = 16.146 (4) Å
β = 97.491 (4)°
*V* = 2595.8 (13) Å^3^
*Z* = 4
Mo *K*α radiation
μ = 1.80 mm^−1^
*T* = 273 (2) K
0.19 × 0.17 × 0.15 mm

#### Data collection

Bruker SMART APEXII CCD area-detector diffractometer
Absorption correction: multi-scan (*SADABS*; Sheldrick, 1996 ▶) *T*_min_ = 0.717, *T*_max_ = 0.766
13610 measured reflections
5065 independent reflections
3393 reflections with *I* > 2σ(*I*)
*R*_int_ = 0.037

#### Refinement

*R*[*F* ^2^ > 2σ(*F* ^2^)] = 0.041
*wR*(*F* ^2^) = 0.109
*S* = 0.99
5065 reflections
329 parameters
10 restraints
H atoms treated by a mixture of independent and constrained refinement
Δρ_max_ = 0.86 e Å^−3^
Δρ_min_ = −0.26 e Å^−3^

### 

Data collection: *APEX2* (Bruker, 2007 ▶); cell refinement: *SAINT-Plus* (Bruker, 2007 ▶); data reduction: *SAINT-Plus*; program(s) used to solve structure: *SHELXS97* (Sheldrick, 2008 ▶); program(s) used to refine structure: *SHELXL97* (Sheldrick, 2008 ▶); molecular graphics: *SHELXTL* (Sheldrick, 2008 ▶); software used to prepare material for publication: *SHELXTL*.

## Supplementary Material

Crystal structure: contains datablocks global, I. DOI: 10.1107/S1600536808028663/hy2151sup1.cif

Structure factors: contains datablocks I. DOI: 10.1107/S1600536808028663/hy2151Isup2.hkl

Additional supplementary materials:  crystallographic information; 3D view; checkCIF report

## Figures and Tables

**Table:** 

Ni1—S1	2.1739 (12)
Ni1—S2	2.1617 (12)
Ni1—S3	2.1732 (12)
Ni1—S4	2.1658 (12)
Ni2—N5	2.164 (3)
Ni2—N6	2.065 (3)
Ni2—N7	2.150 (4)
Ni2—N8	2.145 (3)
Ni2—N9	2.071 (4)
Ni2—N10	2.151 (3)

**Table:** 

S2—Ni1—S4	87.98 (5)
S2—Ni1—S3	168.77 (5)
S4—Ni1—S3	92.72 (4)
S2—Ni1—S1	92.58 (4)
S4—Ni1—S1	170.10 (4)
S3—Ni1—S1	88.65 (4)
N6—Ni2—N9	177.56 (15)
N6—Ni2—N8	98.00 (14)
N9—Ni2—N8	81.73 (14)
N6—Ni2—N7	82.24 (16)
N9—Ni2—N7	95.37 (17)
N8—Ni2—N7	95.96 (15)
N6—Ni2—N10	98.69 (14)
N9—Ni2—N10	81.72 (14)
N8—Ni2—N10	163.04 (16)
N7—Ni2—N10	89.25 (16)
N6—Ni2—N5	81.47 (14)
N9—Ni2—N5	100.96 (15)
N8—Ni2—N5	91.32 (13)
N7—Ni2—N5	162.93 (16)
N10—Ni2—N5	88.20 (14)

**Table 2:** Hydrogen-bond geometry (Å, °)

*D*—H⋯*A*	*D*—H	H⋯*A*	*D*⋯*A*	*D*—H⋯*A*
N5—H5*A*⋯N4^i^	0.86 (3)	2.30 (4)	3.098 (6)	154 (3)
N5—H5*B*⋯N2^ii^	0.86 (3)	2.48 (3)	3.186 (5)	140 (4)
N7—H7*A*⋯N3^iii^	0.86 (4)	2.56 (3)	3.207 (7)	134 (3)
N8—H8*B*⋯N3^iii^	0.86 (3)	2.48 (4)	3.164 (6)	138 (3)
N9—H9*A*⋯N1^iv^	0.86 (2)	2.58 (3)	3.387 (6)	156 (5)
N10—H10*C*⋯N2^ii^	0.87 (3)	2.34 (3)	3.198 (5)	173 (5)

Symmetry codes: (i) 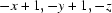; (ii) 
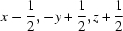
; (iii) 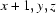; (iv) 
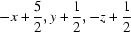
.

**Fig. 1.**
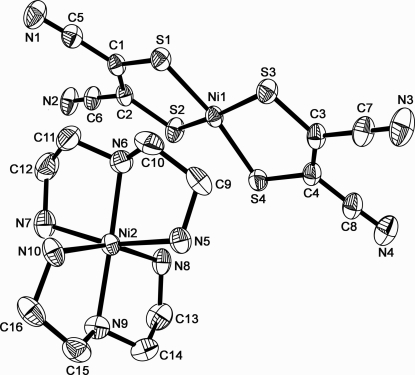
Structure of the title compound. Displacement ellipsoids are drawn at the 30% probability level. H atoms have been omitted for clarity.

## supplementary crystallographic information

### Comment

Bidentate dithiolate ligands form well square-planar complexes with nickel ions in different oxidation states. Due to their unique properties and potential applications in such areas as conducting and magnetic materials, nearinfrared dyes, nonlinear optical materials (Robertson & Cronin, 2002), the ion-pair complexes formed from [M(mnt)_2_]^n-^ (M = Ni, Pd, Pt or Cu) and transition metal complex cations have been intensively studied (Bois *et al.*,  1998; Miller *et al.*,  1989; Ren *et al.*,  2001). We report here a new ion-pair complex.

The title compound is composed of a [Ni(deta)_2_]^2+^ cation and a [Ni(mnt)_2_]^2-^ anion [deta = diethylenetriamine; mnt = maleonitriledithiolate(2-)] (Fig. 1). In the cation, the Ni^II^ ion has a slightly distorted octahedral geometry, formed by six N atoms from two deta ligands, with the Ni—N distances in a range from 2.065 (3) to 2.164 (3) Å (Table 1), which are consistent with the corresponding values in [Ni(en)_3_][Ni(mnt)_2_] (en = ethylenediamine) (Keum *et al.*,  1992). The Ni^II^ ion in the anion is four-coordinated by four S atoms and these five atoms form a square plane with a mean deviation of 0.161 (6) Å. The Ni—S bond lengths [2.1617 (12)–2.1739 (12) Å] are also in agreement with those found in the above complex. The cations and anions are connected by N—H···N hydrogen bonds (Table 2).

### Experimental

The synthesis procedure of the title compound was as following: Ni(ClO_4_)_2_.6H_2_O (0.037 g, 0.10 mmol) was dissolved in methanol (10 ml) at room temperature with stirring and then deta (0.021 g, 0.20 mmol) was added. A solution of Na_2_[Ni(mnt)_2_] (0.033 g, 0.10 mmol) (Simmons *et al.*,  1962) in methanol (10 ml) was slowly added to the above solution and the mixture was stirred for another 30 min. After filtering, the filtrate was undisturbed for about two weeks at room temperature in air to produce blue crystals suitable for X-ray diffraction (yield 61.75%, 0.037 g). Analysis, calculated for C_16_H_26_N_10_Ni_2_S_4_: C 31.81, H 4.34, N 23.19%; found: C 31.76, H 4.31, N 23.24%.

### Refinement

H atoms bound to N atoms were found in difference Fourier maps and refined isotropically, with a restraint of N—H = 0.86 (1) Å. H atoms bound to C atoms were positioned geometrically and refined as riding atoms, with C—H = 0.97 Å and *U*_iso_(H) = 1.2*U*_eq_(C).

### Crystal data

**Table:**

[Ni(C_4_H_13_N_3_)_2_][Ni(C_4_N_2_S_2_)_2_]	*F*(000) = 1248
*M**_r_* = 604.13	*D*_x_ = 1.546 Mg m^−^^3^
Monoclinic, *P*2_1_/*n*	Mo *K*α radiation, λ = 0.71073 Å
Hall symbol: -P 2yn	Cell parameters from 3163 reflections
*a* = 9.589 (3) Å	θ = 2.4–23.8°
*b* = 16.910 (5) Å	µ = 1.80 mm^−^^1^
*c* = 16.146 (4) Å	*T* = 273 K
β = 97.491 (4)°	Block, blue
*V* = 2595.8 (13) Å^3^	0.19 × 0.17 × 0.15 mm
*Z* = 4	

### Data collection

**Table:**

Bruker SMART APEXII CCD area-detector diffractometer	5065 independent reflections
Radiation source: fine-focus sealed tube	3393 reflections with *I* > 2σ(*I*)
graphite	*R*_int_ = 0.037
φ and ω scans	θ_max_ = 26.0°, θ_min_ = 2.3°
Absorption correction: multi-scan (SADABS; Sheldrick, 1996)	*h* = −11→11
*T*_min_ = 0.717, *T*_max_ = 0.766	*k* = −20→19
13610 measured reflections	*l* = −17→19

### Refinement

**Table:**

Refinement on *F*^2^	Primary atom site location: structure-invariant direct methods
Least-squares matrix: full	Secondary atom site location: difference Fourier map
*R*[*F*^2^ > 2σ(*F*^2^)] = 0.041	Hydrogen site location: inferred from neighbouring sites
*wR*(*F*^2^) = 0.109	H atoms treated by a mixture of independent and constrained refinement
*S* = 0.99	*w* = 1/[σ^2^(*F*_o_^2^) + (0.058*P*)^2^] where *P* = (*F*_o_^2^ + 2*F*_c_^2^)/3
5065 reflections	(Δ/σ)_max_ < 0.001
329 parameters	Δρ_max_ = 0.86 e Å^−^^3^
10 restraints	Δρ_min_ = −0.26 e Å^−^^3^

### Fractional atomic coordinates and isotropic or equivalent isotropic displacement parameters (Å^2^)

**Table:**

	*x*	*y*	*z*	*U*_iso_*/*U*_eq_	
Ni1	0.72467 (5)	0.23459 (3)	0.01993 (3)	0.04722 (16)	
Ni2	0.97276 (5)	0.40148 (3)	0.25321 (3)	0.04194 (15)	
S1	0.78132 (10)	0.13475 (6)	0.10229 (6)	0.0555 (3)	
S2	0.90403 (11)	0.21706 (7)	−0.04639 (7)	0.0638 (3)	
S3	0.52234 (11)	0.23887 (7)	0.06673 (7)	0.0598 (3)	
S4	0.68871 (10)	0.34610 (6)	−0.04535 (7)	0.0576 (3)	
N1	1.0808 (4)	0.0021 (3)	0.1686 (3)	0.0828 (12)	
N2	1.2281 (4)	0.0992 (2)	−0.0397 (2)	0.0727 (11)	
N3	0.1991 (4)	0.3603 (3)	0.0519 (3)	0.0980 (15)	
N4	0.3914 (5)	0.4842 (3)	−0.1113 (3)	0.0902 (13)	
N5	0.7588 (4)	0.4323 (2)	0.2690 (2)	0.0532 (8)	
N6	0.8844 (4)	0.2901 (2)	0.2388 (2)	0.0573 (9)	
N7	1.1630 (4)	0.3364 (3)	0.2450 (3)	0.0670 (10)	
N8	0.9396 (4)	0.4370 (2)	0.1246 (2)	0.0551 (9)	
N9	1.0692 (4)	0.5111 (2)	0.2663 (2)	0.0576 (9)	
N10	1.0222 (4)	0.4021 (3)	0.3870 (2)	0.0552 (9)	
C1	0.9425 (4)	0.1059 (2)	0.0742 (2)	0.0487 (9)	
C2	0.9942 (4)	0.1410 (2)	0.0098 (2)	0.0498 (9)	
C3	0.4468 (4)	0.3230 (2)	0.0193 (2)	0.0546 (10)	
C4	0.5168 (4)	0.3686 (2)	−0.0311 (2)	0.0532 (10)	
C5	1.0189 (4)	0.0466 (3)	0.1249 (3)	0.0587 (11)	
C6	1.1252 (4)	0.1169 (3)	−0.0162 (2)	0.0564 (11)	
C7	0.3083 (5)	0.3441 (3)	0.0363 (3)	0.0676 (12)	
C8	0.4494 (5)	0.4331 (3)	−0.0751 (3)	0.0656 (12)	
C9	0.6713 (4)	0.3606 (3)	0.2543 (3)	0.0690 (12)	
H9C	0.6357	0.3564	0.1954	0.083*	
H9B	0.5916	0.3639	0.2854	0.083*	
C10	0.7577 (5)	0.2887 (3)	0.2815 (3)	0.0711 (13)	
H10A	0.7841	0.2894	0.3416	0.085*	
H10B	0.7039	0.2410	0.2668	0.085*	
C11	0.9946 (5)	0.2337 (3)	0.2698 (3)	0.0759 (14)	
H11A	0.9634	0.1802	0.2563	0.091*	
H11B	1.0161	0.2381	0.3301	0.091*	
C12	1.1225 (6)	0.2523 (3)	0.2293 (3)	0.0850 (16)	
H12A	1.1994	0.2181	0.2518	0.102*	
H12B	1.1030	0.2429	0.1696	0.102*	
C13	1.0004 (5)	0.5157 (3)	0.1168 (3)	0.0748 (13)	
H13A	0.9436	0.5450	0.0730	0.090*	
H13B	1.0944	0.5106	0.1013	0.090*	
C14	1.0069 (5)	0.5602 (3)	0.1974 (3)	0.0748 (13)	
H14A	1.0627	0.6078	0.1945	0.090*	
H14B	0.9129	0.5758	0.2066	0.090*	
C15	1.0661 (5)	0.5377 (3)	0.3519 (3)	0.0694 (12)	
H15A	0.9721	0.5556	0.3585	0.083*	
H15B	1.1303	0.5818	0.3639	0.083*	
C16	1.1076 (5)	0.4719 (3)	0.4112 (2)	0.0704 (13)	
H16A	1.2063	0.4595	0.4109	0.084*	
H16B	1.0943	0.4879	0.4674	0.084*	
H8A	0.8544 (16)	0.441 (2)	0.101 (2)	0.051 (11)*	
H8B	0.971 (4)	0.4005 (18)	0.095 (2)	0.073 (15)*	
H7A	1.219 (4)	0.352 (3)	0.211 (2)	0.090 (17)*	
H10C	0.944 (3)	0.406 (3)	0.408 (3)	0.091 (17)*	
H5A	0.727 (5)	0.4692 (19)	0.235 (2)	0.093 (18)*	
H6A	0.860 (4)	0.281 (2)	0.1865 (8)	0.055 (12)*	
H7B	1.217 (4)	0.341 (3)	0.2916 (17)	0.12 (2)*	
H10D	1.066 (6)	0.362 (3)	0.409 (4)	0.16 (3)*	
H9A	1.1581 (18)	0.503 (4)	0.267 (4)	0.16 (3)*	
H5B	0.769 (5)	0.448 (3)	0.3200 (12)	0.101 (18)*	

### Atomic displacement parameters (Å^2^)

**Table:**

	*U*^11^	*U*^22^	*U*^33^	*U*^12^	*U*^13^	*U*^23^
Ni1	0.0377 (3)	0.0536 (3)	0.0497 (3)	0.0057 (2)	0.0033 (2)	−0.0112 (2)
Ni2	0.0429 (3)	0.0502 (3)	0.0324 (2)	−0.0003 (2)	0.00373 (19)	−0.0015 (2)
S1	0.0412 (5)	0.0638 (7)	0.0642 (6)	0.0081 (5)	0.0164 (5)	−0.0001 (5)
S2	0.0514 (6)	0.0822 (8)	0.0599 (6)	0.0189 (6)	0.0160 (5)	0.0104 (6)
S3	0.0503 (6)	0.0652 (7)	0.0663 (7)	0.0126 (5)	0.0161 (5)	−0.0012 (5)
S4	0.0451 (6)	0.0601 (7)	0.0667 (7)	0.0028 (5)	0.0040 (5)	−0.0026 (5)
N1	0.062 (2)	0.098 (3)	0.093 (3)	0.027 (2)	0.024 (2)	0.027 (2)
N2	0.055 (2)	0.110 (3)	0.055 (2)	0.029 (2)	0.0172 (18)	0.016 (2)
N3	0.078 (3)	0.134 (4)	0.089 (3)	0.048 (3)	0.038 (2)	0.009 (3)
N4	0.097 (3)	0.094 (3)	0.077 (3)	0.032 (3)	0.000 (2)	0.008 (2)
N5	0.048 (2)	0.067 (2)	0.044 (2)	0.0015 (18)	0.0033 (16)	−0.0034 (19)
N6	0.067 (2)	0.056 (2)	0.047 (2)	−0.0026 (18)	−0.0011 (18)	−0.0019 (17)
N7	0.055 (2)	0.087 (3)	0.059 (2)	0.014 (2)	0.008 (2)	−0.003 (2)
N8	0.048 (2)	0.077 (3)	0.0393 (19)	0.014 (2)	0.0049 (16)	0.0003 (18)
N9	0.065 (2)	0.059 (2)	0.051 (2)	−0.0045 (19)	0.0138 (17)	−0.0004 (17)
N10	0.042 (2)	0.087 (3)	0.0367 (17)	−0.009 (2)	0.0043 (15)	−0.0018 (18)
C1	0.037 (2)	0.058 (3)	0.051 (2)	0.0068 (18)	0.0077 (17)	−0.0086 (19)
C2	0.037 (2)	0.064 (3)	0.049 (2)	0.0069 (19)	0.0053 (17)	−0.0067 (19)
C3	0.048 (2)	0.068 (3)	0.047 (2)	0.017 (2)	0.0024 (18)	−0.016 (2)
C4	0.050 (2)	0.058 (3)	0.049 (2)	0.013 (2)	−0.0017 (18)	−0.013 (2)
C5	0.043 (2)	0.073 (3)	0.063 (3)	0.007 (2)	0.020 (2)	0.002 (2)
C6	0.051 (2)	0.076 (3)	0.043 (2)	0.016 (2)	0.0078 (18)	0.006 (2)
C7	0.064 (3)	0.087 (3)	0.053 (3)	0.025 (3)	0.014 (2)	−0.003 (2)
C8	0.063 (3)	0.077 (3)	0.054 (3)	0.018 (2)	−0.003 (2)	−0.005 (2)
C9	0.048 (2)	0.092 (4)	0.067 (3)	−0.018 (3)	0.006 (2)	−0.014 (3)
C10	0.073 (3)	0.070 (3)	0.071 (3)	−0.026 (3)	0.014 (2)	−0.002 (2)
C11	0.093 (4)	0.057 (3)	0.073 (3)	0.009 (3)	−0.009 (3)	0.008 (2)
C12	0.096 (4)	0.072 (4)	0.082 (3)	0.032 (3)	−0.005 (3)	−0.009 (3)
C13	0.090 (3)	0.081 (4)	0.056 (3)	0.004 (3)	0.021 (2)	0.023 (2)
C14	0.087 (4)	0.052 (3)	0.085 (3)	−0.001 (2)	0.012 (3)	0.008 (2)
C15	0.070 (3)	0.067 (3)	0.071 (3)	−0.005 (2)	0.010 (2)	−0.028 (2)
C16	0.066 (3)	0.100 (4)	0.045 (2)	−0.025 (3)	0.006 (2)	−0.013 (2)

### Geometric parameters (Å, °)

**Table:**

Ni1—S1	2.1739 (12)	N9—C15	1.456 (5)
Ni1—S2	2.1617 (12)	N9—H9A	0.86 (3)
Ni1—S3	2.1732 (12)	N10—C16	1.461 (5)
Ni1—S4	2.1658 (12)	N10—H10C	0.87 (3)
Ni2—N5	2.164 (3)	N10—H10D	0.85 (6)
Ni2—N6	2.065 (3)	C1—C2	1.347 (5)
Ni2—N7	2.150 (4)	C1—C5	1.434 (6)
Ni2—N8	2.145 (3)	C2—C6	1.434 (5)
Ni2—N9	2.071 (4)	C3—C4	1.359 (6)
Ni2—N10	2.151 (3)	C3—C7	1.436 (5)
S1—C1	1.737 (4)	C4—C8	1.412 (6)
S2—C2	1.738 (4)	C9—C10	1.505 (6)
S3—C3	1.730 (4)	C9—H9C	0.9700
S4—C4	1.737 (4)	C9—H9B	0.9700
N1—C5	1.144 (5)	C10—H10A	0.9700
N2—C6	1.143 (5)	C10—H10B	0.9700
N3—C7	1.141 (5)	C11—C12	1.497 (7)
N4—C8	1.146 (5)	C11—H11A	0.9700
N5—C9	1.477 (5)	C11—H11B	0.9700
N5—H5A	0.86 (3)	C12—H12A	0.9700
N5—H5B	0.86 (3)	C12—H12B	0.9700
N6—C11	1.463 (5)	C13—C14	1.497 (6)
N6—C10	1.473 (5)	C13—H13A	0.9700
N6—H6A	0.86 (2)	C13—H13B	0.9700
N7—C12	1.486 (6)	C14—H14A	0.9700
N7—H7A	0.86 (4)	C14—H14B	0.9700
N7—H7B	0.86 (4)	C15—C16	1.488 (6)
N8—C13	1.466 (6)	C15—H15A	0.9700
N8—H8A	0.86 (3)	C15—H15B	0.9700
N8—H8B	0.86 (3)	C16—H16A	0.9700
N9—C14	1.453 (5)	C16—H16B	0.9700
			
S2—Ni1—S4	87.98 (5)	C5—C1—S1	117.0 (3)
S2—Ni1—S3	168.77 (5)	C1—C2—C6	121.7 (4)
S4—Ni1—S3	92.72 (4)	C1—C2—S2	121.4 (3)
S2—Ni1—S1	92.58 (4)	C6—C2—S2	116.9 (3)
S4—Ni1—S1	170.10 (4)	C4—C3—C7	121.0 (4)
S3—Ni1—S1	88.65 (4)	C4—C3—S3	121.4 (3)
N6—Ni2—N9	177.56 (15)	C7—C3—S3	117.7 (3)
N6—Ni2—N8	98.00 (14)	C3—C4—C8	120.7 (4)
N9—Ni2—N8	81.73 (14)	C3—C4—S4	120.5 (3)
N6—Ni2—N7	82.24 (16)	C8—C4—S4	118.8 (3)
N9—Ni2—N7	95.37 (17)	N1—C5—C1	176.5 (5)
N8—Ni2—N7	95.96 (15)	N2—C6—C2	177.4 (4)
N6—Ni2—N10	98.69 (14)	N3—C7—C3	178.2 (5)
N9—Ni2—N10	81.72 (14)	N4—C8—C4	178.1 (5)
N8—Ni2—N10	163.04 (16)	N5—C9—C10	109.7 (3)
N7—Ni2—N10	89.25 (16)	N5—C9—H9C	109.7
N6—Ni2—N5	81.47 (14)	C10—C9—H9C	109.7
N9—Ni2—N5	100.96 (15)	N5—C9—H9B	109.7
N8—Ni2—N5	91.32 (13)	C10—C9—H9B	109.7
N7—Ni2—N5	162.93 (16)	H9C—C9—H9B	108.2
N10—Ni2—N5	88.20 (14)	N6—C10—C9	107.8 (3)
C1—S1—Ni1	102.61 (14)	N6—C10—H10A	110.1
C2—S2—Ni1	102.53 (13)	C9—C10—H10A	110.1
C3—S3—Ni1	102.40 (15)	N6—C10—H10B	110.1
C4—S4—Ni1	102.68 (15)	C9—C10—H10B	110.1
C9—N5—Ni2	108.1 (3)	H10A—C10—H10B	108.5
C9—N5—H5A	110 (3)	N6—C11—C12	108.0 (4)
Ni2—N5—H5A	111 (3)	N6—C11—H11A	110.1
C9—N5—H5B	114 (3)	C12—C11—H11A	110.1
Ni2—N5—H5B	101 (3)	N6—C11—H11B	110.1
H5A—N5—H5B	112 (5)	C12—C11—H11B	110.1
C11—N6—C10	115.5 (4)	H11A—C11—H11B	108.4
C11—N6—Ni2	106.8 (3)	N7—C12—C11	109.7 (4)
C10—N6—Ni2	108.3 (3)	N7—C12—H12A	109.7
C11—N6—H6A	108 (3)	C11—C12—H12A	109.7
C10—N6—H6A	109 (3)	N7—C12—H12B	109.7
Ni2—N6—H6A	109 (3)	C11—C12—H12B	109.7
C12—N7—Ni2	107.2 (3)	H12A—C12—H12B	108.2
C12—N7—H7A	110 (3)	N8—C13—C14	110.7 (3)
Ni2—N7—H7A	120 (3)	N8—C13—H13A	109.5
C12—N7—H7B	110 (4)	C14—C13—H13A	109.5
Ni2—N7—H7B	109 (4)	N8—C13—H13B	109.5
H7A—N7—H7B	100 (5)	C14—C13—H13B	109.5
C13—N8—Ni2	109.1 (3)	H13A—C13—H13B	108.1
C13—N8—H8A	105 (3)	N9—C14—C13	110.2 (4)
Ni2—N8—H8A	118 (2)	N9—C14—H14A	109.6
C13—N8—H8B	116 (3)	C13—C14—H14A	109.6
Ni2—N8—H8B	108 (3)	N9—C14—H14B	109.6
H8A—N8—H8B	101 (4)	C13—C14—H14B	109.6
C14—N9—C15	119.5 (4)	H14A—C14—H14B	108.1
C14—N9—Ni2	107.4 (3)	N9—C15—C16	110.1 (4)
C15—N9—Ni2	108.0 (3)	N9—C15—H15A	109.6
C14—N9—H9A	114 (4)	C16—C15—H15A	109.6
C15—N9—H9A	100 (4)	N9—C15—H15B	109.6
Ni2—N9—H9A	107 (5)	C16—C15—H15B	109.6
C16—N10—Ni2	108.4 (3)	H15A—C15—H15B	108.2
C16—N10—H10C	108 (3)	N10—C16—C15	109.8 (3)
Ni2—N10—H10C	108 (3)	N10—C16—H16A	109.7
C16—N10—H10D	108 (5)	C15—C16—H16A	109.7
Ni2—N10—H10D	117 (5)	N10—C16—H16B	109.7
H10C—N10—H10D	107 (5)	C15—C16—H16B	109.7
C2—C1—C5	122.5 (3)	H16A—C16—H16B	108.2
C2—C1—S1	120.5 (3)		

### Hydrogen-bond geometry (Å, °)

**Table:**

*D*—H···*A*	*D*—H	H···*A*	*D*···*A*	*D*—H···*A*
N5—H5A···N4^i^	0.86 (3)	2.30 (4)	3.098 (6)	154 (3)
N5—H5B···N2^ii^	0.86 (3)	2.48 (3)	3.186 (5)	140 (4)
N7—H7A···N3^iii^	0.86 (4)	2.56 (3)	3.207 (7)	134 (3)
N8—H8B···N3^iii^	0.86 (3)	2.48 (4)	3.164 (6)	138 (3)
N9—H9A···N1^iv^	0.86 (2)	2.58 (3)	3.387 (6)	156 (5)
N10—H10C···N2^ii^	0.87 (3)	2.34 (3)	3.198 (5)	173 (5)

Symmetry codes: (i) −*x*+1, −*y*+1, −*z*; (ii) *x*−1/2, −*y*+1/2, *z*+1/2; (iii) *x*+1, *y*, *z*; (iv) −*x*+5/2, *y*+1/2, −*z*+1/2.
